# Supplementary material for: Seminoma and Embryonal Carcinoma Footprints Identified by Analysis of Integrated Genome-Wide Epigenetic and Expression Profiles of Germ Cell Cancer Cell Lines
Source: PLoS One. 2014 Jun 2;9(6):e98330. doi: 10.1371/journal.pone.0098330 (PMC4041891; doi:10.1371/journal.pone.0098330)
Supplement: File S1 — ZIP file containing DMRforPairs output for significant regions. Please start from the html files. (ZIP) [file pone.0098330.s008.zip › figures/31610.pdf]

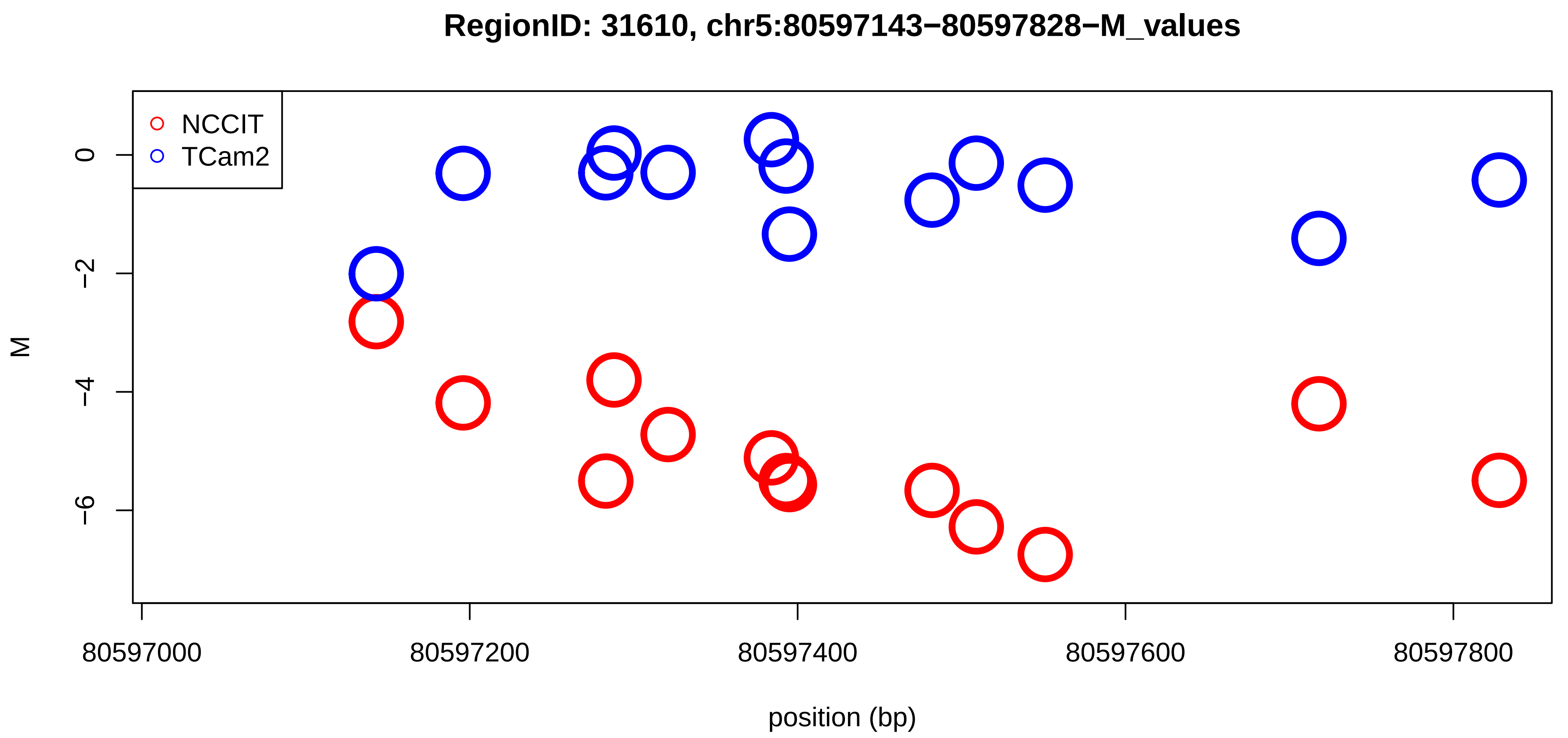
RegionID: 31610, chr5:80597143−80597828−M_values
NCCIT
TCam2
M
0
−2
−4
−6
80597000
80597200
80597400
80597600
80597800
position (bp)

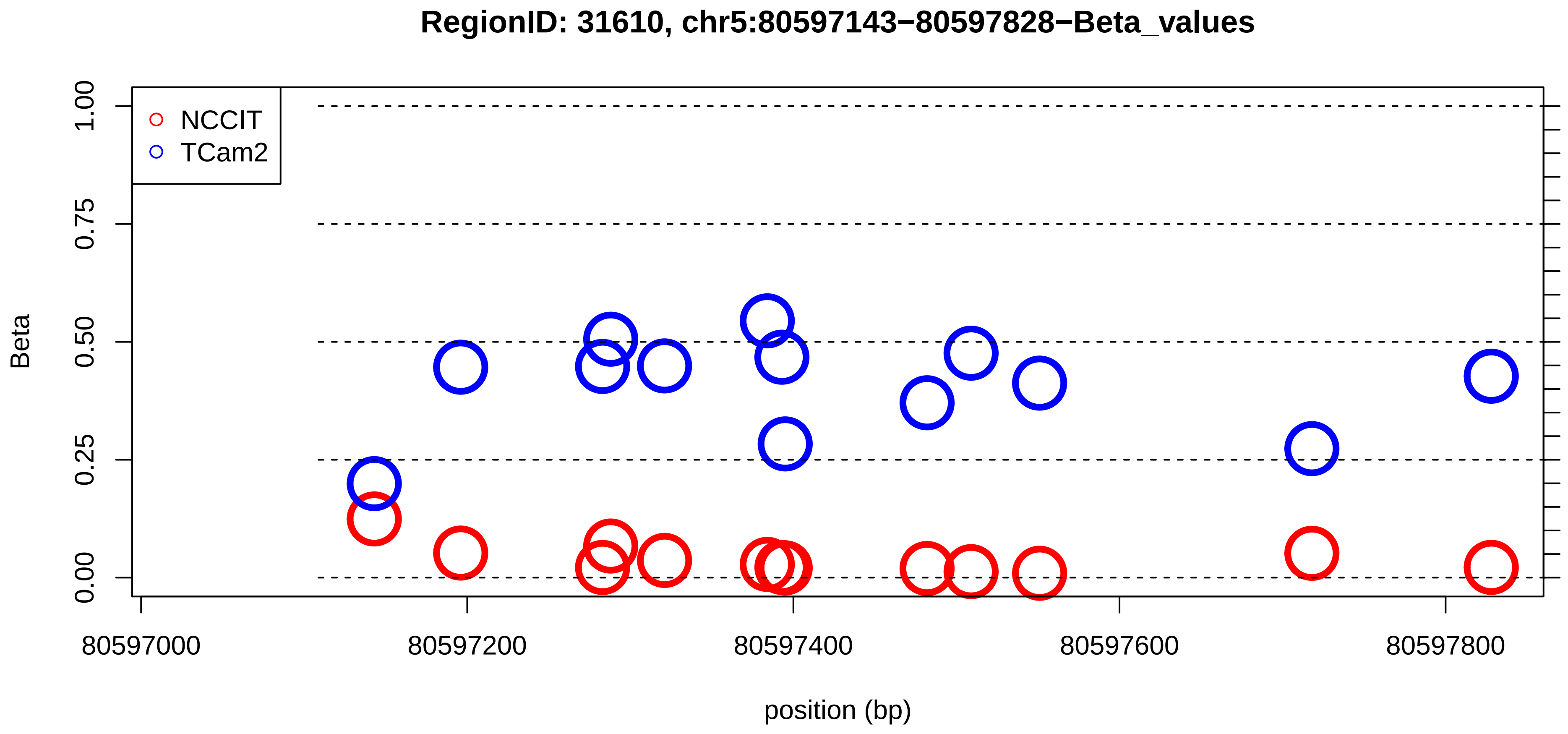
RegionID: 31610, chr5:80597143−80597828−Beta_values
NCCIT
TCam2
Beta
1.00
0.75
0.50
0.25
0.00
80597000
80597200
80597400
80597600
80597800
position (bp)

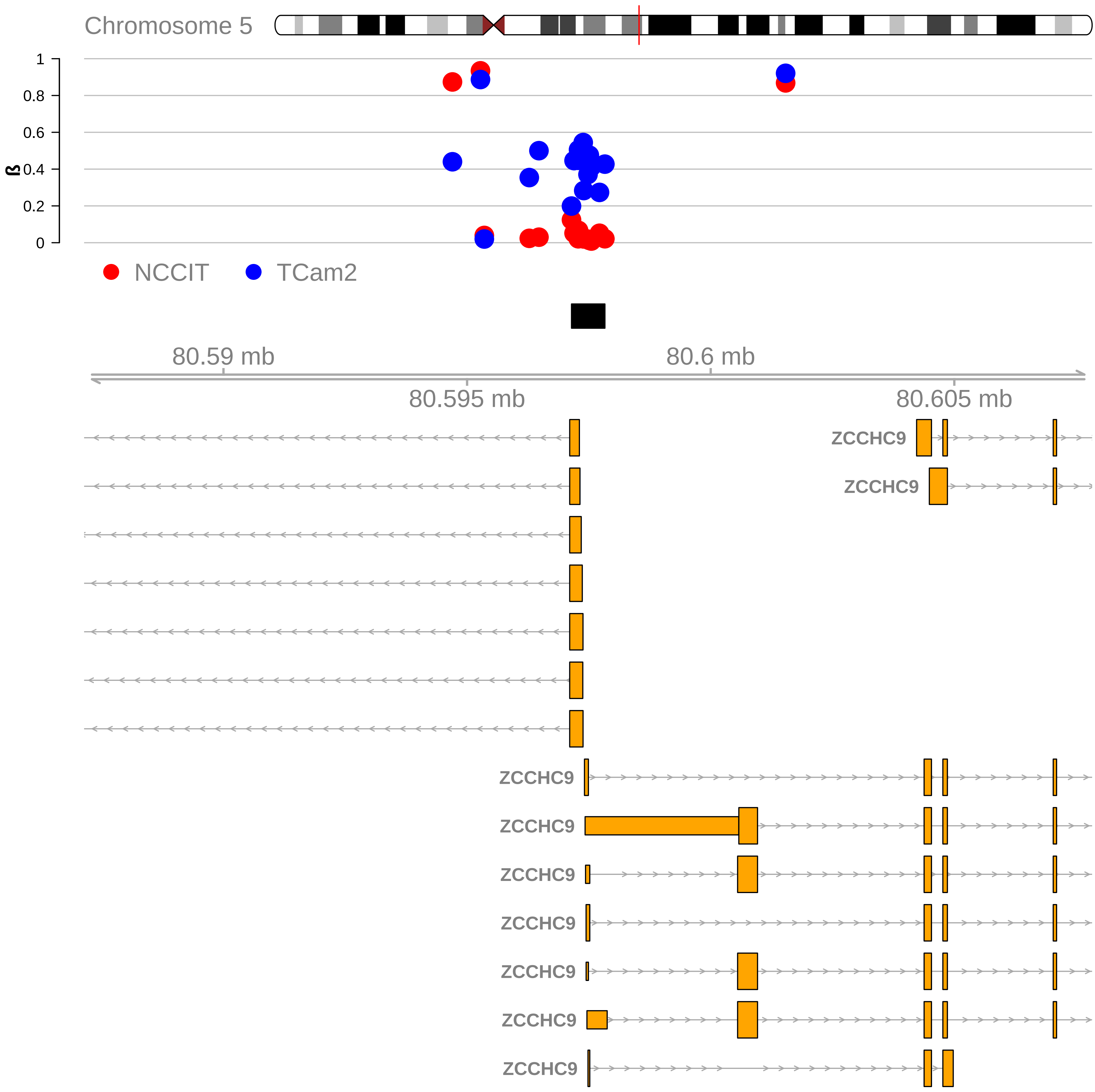

Chromosome 5
1
0.8
0.6
ß
0.4
0.2
0
NCCIT
TCam2
80.59 mb
80.6 mb
80.595 mb
80.605 mb
ZCCHC9
ZCCHC9
ZCCHC9
ZCCHC9
ZCCHC9
ZCCHC9
ZCCHC9
ZCCHC9
ZCCHC9
